# Can neoadjuvant endocrine therapy de-escalate surgery in luminal breast cancer? A four-year retrospective study from a single referral center

**DOI:** 10.3389/fonc.2026.1831764

**Published:** 2026-07-28

**Authors:** Aleksander Skulimowski, Jakub Skórniak, Tomasz Michalik, Marcin Ekiert, Karolina Skulimowska, Piotr Kasprzak, Rafał Matkowski

**Affiliations:** 1 Breast Unit, II Department of Surgical Oncology, Lower Silesian Center of Oncology, Pulmonology, and Hematology, Wroclaw, Poland; 2 Department of Oncology, Faculty of Medicine, Wroclaw Medical University, Wroclaw, Poland; 3 Department of Oncology, Lower Silesian Center of Oncology, Pulmonology, and Hematology, Wroclaw, Poland; 4 Department of Endocrinology, Diabetology and Internal Medicine, Tadeusz Marciniak Lower Silesia Specialist Hospital-Centre for Medical Emergency, Wroclaw, Poland; 5 Department of Radiology, Lower Silesian Center of Oncology, Pulmonology, and Hematology, Wroclaw, Poland

**Keywords:** breast cancer, breast surgery, de-escalation of axillary surgery, endocrine therapy, neoadjuvant therapy, tumor reduction

## Abstract

**Introduction:**

Postmenopausal women commonly present with HR-positive/HER2-negative breast cancer. Neoadjuvant endocrine therapy (NET) may facilitate breast-conserving surgery (BCS) and de-escalation of axillary surgery. This study evaluated NET efficacy in enabling surgical de-escalation in luminal breast cancer.

**Materials and methods:**

A retrospective study included postmenopausal women (≥49 years) with newly diagnosed, luminal breast cancer treated with NET between May 2021 and May 2024 at a single tertiary breast unit. Patients with stage IV disease or contraindications to surgery were excluded. Clinical and pathologic variables were collected from chart review. Tumor and nodal downstaging were defined by decrease of at least one level from clinical (cT or cN) to pathologic (ypT or ypN) stage. Multivariable logistic regression was used to identify predictors of downstaging.

**Results:**

A total of 124 patients (128 tumors) were included. Mean age at diagnosis was 69 years. NET resulted in a significant reduction in tumor size (mean absolute reduction 10.93 mm; p < 0.0001; mean relative reduction 23.4%; p < 0.0001). BCS was achieved in 75.8% of cases, and NET was estimated to have avoided mastectomy in at least 27 patients. Lobular histology (OR 2.92, 95% CI 1.20–7.00; p = 0.017) and absence of clinical nodal involvement (OR 3.10, 95% CI 1.45–6.55; p = 0.003) were independently associated with T-stage downstaging.

Regarding axillary management, 75.8% underwent sentinel lymph node biopsy and 22.7% upfront axillary lymph node dissection. Among patients initially managed with SLNB, 35% required completion ALND. Of 60 clinically node-positive (cN1) patients, 8 (12.7%) achieved ypN0 status after NET. Micrometastatic residual disease was observed in 4 additional cases (6.3% of cN1 patients).

**Conclusions:**

NET effectively facilitates breast-conserving surgery in postmenopausal patients with luminal breast cancer. However, its impact on achieving nodal clearance and reducing axillary dissection remains limited.

## 1 Introduction

Breast cancer is the most common malignancy among women, with approximately two million new cases diagnosed worldwide each year (1). It accounts for 11,6% of all new cancer diagnoses worldwide, and incidence rates have been rising steadily over the past two decades (2). According to the study by National Cancer Registry, breast cancer is also the most common cancer in female Polish population, accounting for 24% of all new cases. It is the second leading cause of cancer-related mortality of females in Poland (3). Hormone receptor–positive, HER2-negative (HR+/HER2−) tumors breast cancer, comprise roughly 70% of cases (4–6), making this subtype a critical focus of ongoing research.

Early-stage HR+/HER2− breast cancer generally carries a favorable prognosis (7–9), yet its response to neoadjuvant chemotherapy (NAC) is modest, particularly in terms of nodal downstaging (10). Data from the I-SPY trial and the ACOSOG Z1071 study show that patients with this subtype achieve significantly lower nodal pathological complete response (pCR) rates compared with those who have HER2-positive or triple-negative tumors (11, 12). While tumor burden reduction—especially achieving local pCR—is associated with improved outcomes, NAC remains the preferred approach in cases of large primary tumors and/or nodal involvement especially in premenopausal women (13–15).

However, NAC is accompanied by systemic toxicity, and its limited efficacy in HR+/HER2− disease complicates treatment decisions (16–19). Moreover, no validated histopathological or molecular predictors of NAC response are currently available, although several studies are under way (20, 21).

Neoadjuvant endocrine therapy (NET) has emerged as a potential alternative for patients with locally advanced HR+/HER2− breast cancer. Initially reserved for those unfit for NAC or ineligible for immediate surgery, NET is now supported by evidence showing that sufficient treatment duration, with most studies showing an appropriate duration of NET of 6–9 months, can enable breast-conserving surgery (BCS) in up to 87,5% of cases (22–25). Nonetheless, while NET often achieves meaningful local tumor reduction, nodal pCR rates remain modest. A meta-analysis comparing NET and NAC reported nodal pCR rates of 8.9% and 14.9%, respectively in HR+/HER2 (26).

Given its low toxicity profile, NET may have a role in converting clinically node-positive disease to node-negative status. Yet current recommendations for managing residual nodal disease after NET are extrapolated from NAC data rather than derived from high-quality, NET-specific trials (27). In practice, many centers manage the axilla after NET in the same manner as in patients undergoing upfront surgery, resulting in lower rates of axillary lymph node dissection (ALND) compared with the post-NAC setting (28–30). Some studies suggest that omitting ALND in selected patients does not compromise oncologic outcomes (31), but robust evidence on optimal axillary management following NET is lacking.

Given the current body of evidence, in the present study we aimed to retrospectively evaluate the efficacy of NET in de-escalation of both breast and nodal surgery in the luminal cancers.

## 2 Materials and methods

### 2.1 Study design and patient selection

We conducted a retrospective review of female patients treated at the Breast Unit of the Lower Silesian Center of Oncology, Pulmonology, and Hematology between May 2021 and May 2024. Eligible patients were consecutive postmenopausal women (=> 49 years old), who had newly diagnosed, estrogen receptor–positive (ER+), progesterone receptor–positive (PR+), and HER2-negative invasive breast cancer.

Estrogen receptor (ER), progesterone receptor (PR), and HER2 status were assessed on formalin-fixed, paraffin-embedded tissue specimens obtained from vacuum-assisted biopsy and/or surgical specimens. All analyses were performed by the Department of Pathology and were based on immunohistochemical staining.

ER and PR positivity were defined as nuclear staining in ≥1% of tumor cells, according to standard ASCO/CAP guidelines (32). HER2 status was determined by immunohistochemistry and, when equivocal (2+), confirmed by *in situ* hybridization. HER2-negative status was defined as IHC 0/1+ or IHC 2+ without gene amplification.

All receptor statuses were extracted from the official pathology reports included in the institutional medical records and used for study classification and subsequent analyses.

We excluded patients with:

- stage IV disease at diagnosis,
- recurrent breast cancer, or
- contraindications to surgical treatment.

A total of 124 patients met the inclusion criteria; four presented with synchronous bilateral luminal cancers. All underwent complete diagnostic work-up at our institution.

At presentation, all patients underwent bilateral diagnostic mammography and breast and axillary nodes ultrasound. Vacuum-assisted biopsy (VAB) of breast lesion/s, performed under ultrasound or stereotactic (MRI or MMG) guidance, was used to confirm malignancy. Standard laboratory tests and posteroanterior chest radiography were performed in all cases.

Clinical nodal positivity (cN1) was defined based on axillary ultrasound findings performed by dedicated breast radiologists. Lymph nodes were considered metastatic in the presence of morphological abnormalities, including cortical thickening (typically >3 mm), loss of fatty hilum, rounded configuration, and/or abnormal vascularity (33, 34).

All lesions targeted for surgery were marked with metal clips during biopsy or prior to the initiation of neoadjuvant endocrine therapy. Axillary lymph nodes were not clipped or sampled, and clinical nodal status was assessed based on imaging findings only.

Breast magnetic resonance imaging (MRI) was routinely obtained in invasive lobular carcinoma and selectively in cases of multifocal disease. Additional imaging (e.g., abdominal ultrasound, chest or abdominal computed tomography) was ordered at the discretion of the treating physician.

The decision to initiate neoadjuvant endocrine therapy (NET) was made by a multidisciplinary breast cancer tumor board.

We collected the following variables:

1. age at diagnosis.
2. tumor laterality.
3. radiological tumor size at presentation and prior to surgery; multifocal tumors were measured by summing the two largest lesions. In case of size discrepancy between mammography and ultrasound, the larger measurement was recorded. MRI measurements were excluded from statistical analysis due to selective use.
4. histopathological tumor size from surgical specimens; multifocal tumors were measured as above.
5. type and duration of NET.
6. nodal status on clinical and imaging evaluation.
7. clinical and pathological staging (cTNM, ycTNM, ypTNM), tumor type, grade, and presence of ductal carcinoma *in situ* (DCIS).
8. ER, PR, HER2, and Ki-67 immunohistochemistry results from VAB specimens.
9. ypN stage as defined by nodal metastases: number of micro- and macrometastases.
10. type of breast surgery (BCS or mastectomy) and axillary surgery [sentinel lymph node biopsy (SLNB), axillary lymph node dissection (ALND), or SLNB followed by ALND].

### 2.2 Statistical analysis

Data were analyzed using IBM SPSS Statistics version 26.0 (Armonk, NY, USA).Continuous variables with normal distribution were compared using parametric tests (Student t-test), whereas non-normally distributed variables were analyzed with non-parametric tests (Mann-Whitney test for two independent groups and Kruskal-Wallis test for more than two independent groups). Nominal variables were analyzed using the χ² test. Correlations were evaluated using Spearman’s correlation coefficient. Logistic regression was applied to estimate odds ratios (OR) with 95% confidence intervals (CI) for binary dependent variables. Variables included in the multivariate logistic regression model were those found to be statistically significant in univariate analysis model, as specified in the relevant **Tables 1**, **2**.

**Table 1:** Predictive logistic regression model for T feature downstaging.

Variable	Univariate OR (95%CI)	p	Multivariate OR (95%CI)	p
Lobular histology	3.20 (1.36–7.44)	0.007	2.92 (1.20–7.00)	**0.017**
Absence of clinical nodal metastates	3.24 (1.56–6.72)	0.002	3.10 (1.45–6.55)	**0.003**
Absence of pathological nodal metastates	2.94 (1.40–6.10)	0.004	1.67 (0.66-4.18)	0.273

p values in bold indicate statistically significant results.

**Table 2:** Predictive logistic regression model for nodal response to NET.

Variable	OR (95%CI)	p
PR expression 31%-60%	8 (1,58 – 40,6)	0,012
Lobular histology	2,64 (0,54 – 12,91)	0,231
ER expression < 100%	0,82 (0,09 – 7,6)	0,86
NET duration over study median	0,42 (0,94 – 1,9)	0,26
Grade 1	0,82 (0,09 – 7,6)	0,86
Tumor < cT2	0,95 (0,17 – 5,3)	0,95

Receiver operating characteristic (ROC) curve analysis was used to evaluate the ability of selected continuous variables to predict binary outcomes. The area under the curve (AUC) was calculated, and optimal cut-off values were determined using the Youden index (35).

Statistical significance was set at p < 0,05.

### 2.3 Preoperative imaging and surgical techniques

All breast lesions targeted for surgery were marked with tissue clips before or during NET. One day prior to surgery, MagTrace^®^ (Endomag Ltd., UK) (1 mL) was injected subcutaneously in the periareolar region. On the morning of surgery, lesions were localized under ultrasound and/or stereotactic guidance. Non-palpable lesions and selected microcalcifications were bracketed with guidewires, with placement confirmed by mammography.

For BCS, standard oncoplastic techniques were used. Sentinel lymph nodes were detected using the SentiMag^®^ system (Endomag Ltd., UK). Intraoperative three-dimensional specimen radiography (MOZART^®^ System, Kubtec Medical Imaging, Stratford, CT) was routinely performed to reduce re-excision risk. Additional margin excision or cavity shaving was performed at the surgeon’s discretion.

The qualification for axillary surgery was based on the results of preoperative imaging and physical examination. Patients without clinically positive axillary lymph nodes were scheduled for sentinel lymph node biopsy (SLNB). In cN0 patients who remained clinically node-negative before surgery, retrieval of at least one sentinel lymph node was considered sufficient for axillary staging.

Patients with clinically node-positive disease who converted to ycN0 status after NET were also scheduled for SLNB; however, retrieval of at least three sentinel lymph nodes was required in these cases. If no tracer uptake was identified intraoperatively, the procedure was converted to ALND. Patients with persistent clinically positive axillary disease after neoadjuvant treatment underwent upfront ALND.

Patients with positive sentinel lymph nodes on histopathological examination (ypN+) were generally considered candidates for completion ALND. However, the final decision was discussed individually during a multidisciplinary tumor board meeting and with the patient. Omission of completion ALND was permitted in selected cases with limited nodal burden (e.g., a single micrometastasis or macrometastasis), provided that adjuvant breast and regional nodal radiotherapy was planned.

### 2.4 Follow-up during neoadjuvant endocrine therapy

Patients undergoing neoadjuvant endocrine therapy (NET) were scheduled for regular follow-up visits in the Breast Unit outpatient clinic. Clinical assessment was typically performed after 1, 3, and 6 months of treatment and included breast and axillary ultrasound to evaluate response to therapy.

Neoadjuvant endocrine therapy (NET) was administered for a minimum duration of 6 months according to an internal institutional protocol. The decision to complete or extend treatment beyond this period was individualized and based on clinical and radiological response as well as surgical planning. NET was considered completed once the patient was deemed eligible for definitive surgical treatment.

Upon completion of NET and prior to selecting the surgical strategy for the breast and axilla, all patients underwent comprehensive reassessment, including physical examination, mammography, and ultrasound. The extent of surgery was determined based on the final clinical and radiological evaluation of tumor response, as well as initial disease characteristics.

When assessing tumor response, the largest tumor diameter identified on imaging or physical examination was taken into account. Decisions regarding breast-conserving surgery versus mastectomy, as well as the extent of axillary surgery, were made within a multidisciplinary tumor board, taking into consideration tumor size reduction, nodal status, imaging findings, and patient-related factors. Particular emphasis was placed on optimizing local control while minimizing surgical morbidity.

### 2.5 Definition of particular variables

To evaluate the efficacy of neoadjuvant endocrine therapy (NET), tumor (T) and nodal (N) downstaging were included in the analysis. Downstaging was defined as a reduction of at least one category in ypT and/or ypN stage following systemic treatment compared with baseline clinical staging.

For the analysis of absolute tumor size changes, cases were classified into five categories: (1) tumor progression (>5 mm increase in largest diameter), (2) minor progression (≤5 mm increase), (3) disease stabilization (change within ±5 mm), (4) tumor reduction (≥5 mm decrease), and (5) pathological complete response (pCR), defined as the absence of residual invasive carcinoma in the breast on histopathological examination.

## 3 Results

The study included 124 patients (128 cancers) with a mean age of 69 years. The basic patients’ demographics, tumor clinicopathological data and surgery types are shown in **Table 3**. Fifty-nine patients (47.6%) presented with right-sided tumors, 61 (49.2%) with left-sided tumors, and four (3.2%) had synchronous bilateral disease. There were 96 (75%) cases of either IIA or IIB stage of disease and 72% of cases were ductal invasive carcinoma (see the appropriate section of **Table 3**). Use of neoadjuvant endocrine therapy (NET) increased over the study period—from 17 patients (13.3%) in 2021 to 53 (41.4%) in 2024. The main indications for NET were:

**Table 3:** Basic patients’, clinicopathological tumor data and surgery types.

Data type	Table legend and results
Basic patients’ and tumor data	mean (median) +/- standard deviation [range]
Age	69 (69) +/- 8,2 [49-88]
Initial size [mm]	37 (32) +/- 20 [9-100]
Multifocal disease	11 (8,6%)
Preoperative size [mm]	25,5 (24) +/- 16,5 [0-100]
Histopathological size [mm]	26,05 (24) +/- 17,8 [0-120]
Approx. reduction (%)	23,4% (20) +/- 36,6 [- 100% - + 118,75%]
Reduction [mm]	10,93 (6) +/- 18,5 [- 90 - + 55]
Duration of neoadjuvant endocrine therapy [months]	8 (8) +/- 2,29 [1-16]
Distribution of cancer stage in study population	Absolute number of patients
IA	7
IB	0
IIA	48
IIB	48
IIIA	14
Histopathological features in study population	Absolute number of patients or mean (median) +/- standard deviation ^*^
Invasive ductal carcinoma	92
Invasive lobular carcinoma	34
Other	2
Grade 1	20
Grade 2	103
Grade 3	5
Absence of clinical nodal metastates	65
Absence of pathological nodal metastates	55
ER stain	97,46 (100) +/- 7,86
PR stain	62,6 (80) +/- 37,5
Ki-67 stain	23,6 (20) +/- 15,5
Types of breast and axillary surgery performed	Absolute number of patients (%)
Breast conserving surgery	97 (75,8%)
Mastectomy	31 (24,2%)
Upfront sentinel lymph node biopsy no further axillary surgery	63 (49,2%)
Upfront axillary lymph node dissection	29 (22,6%)
Completion ALND	34 (26,6%)
No axillary procedure	2 (1,6%)

Data is shown as mean (median) +/- standard deviation.

*where applicable.

- facilitation of breast-conserving surgery (BCS): n = 56
- de-escalation of axillary surgery: n = 47
- both BCS facilitation and axillary de-escalation: n = 16
- temporary delay of surgery due to comorbidities or contraindications: n = 5

Mean NET duration was 8 months (range 1–16 months). There were two cases with a shorter duration of neoadjuvant endocrine therapy (1 and 2 months, respectively). In the first case, treatment was discontinued due to early disease progression detected at the first follow-up assessment. In the second case, NET was discontinued because of treatment-related adverse effects that were not tolerated by the patient, leading to therapy discontinuation. All patients except two (treated with anastrozole) received letrozole.

### 3.1 Breast tumor response

NET resulted in a statistically significant tumor size reduction with mean absolute reduction of 10.93 mm (p < 0.0001) and mean percentage reduction in size of 23.4% (p < 0.0001). BCS was thus performed in 75,8% of cases; 24,2% required mastectomy (see the appropriate section of **Table 3**). Retrospective review suggested that NET likely prevented mastectomy in 27 cases (21%). This subgroup showed greater tumor reduction compared with others i.e. absolute reduction of 22,7 mm vs. 7,61 mm and percentage of tumor reduction of 41,26% vs. 18,33% (both p < 0,0001). Patients in whom mastectomy was prevented were older (mean 71,5 vs. 68 years, p = 0,039) and presented with more advanced disease (higher T stage, p < 0.0001; higher overall stage, p = 0,017).

Pathological complete response (pCR) in the breast was observed in one patient (0,8%). Tumor upstaging occurred in two cases (1,6%), but both remained amenable to BCS. Minor tumor progression was observed in 8 cases (6,45%). In addition, tumor size remained stable in 32 cases (25,8%), defined as a reduction of less than 5 mm.

Overall, 62 patients (48,4%) experienced downstaging of the T feature (**Table 4**). This group demonstrated greater reductions in tumor size compared to group of patients which remained in the same category: absolute reduction of 19,25 mm vs. 4,27 mm and percentage reduction: 40,36% vs. 10,13% (both p < 0,001).

**Table 4:** Distribution of T feature in study population.

cT0	cT1a	cT1b	cT1c	cT2	cT3	cT4
0	0	3	19	64	32	10
ycT0	ycT1a	ycT1b	ycT1c	ycT2	ycT3	ycT4
8	1	8	38	60	9	3
ypT0	ypT1a	ypT1b	ypT1c	ypT2	ypT3	ypT4
1	6	7	39	66	6	2

Univariate analysis identified the following factors as associated with higher odds of T downstaging:

- lobular histology (OR 3,20, 95% CI 1,36–7,44; p = 0,007)
- absence of clinical nodal metastases (OR 3,24, 95% CI 1,56–6,72; p = 0,002)
- absence of pathological nodal metastases (OR 2,94, 95% CI 1,40–6,10; p = 0,004)

On multivariate analysis, lobular histology (OR 2,92, 95% CI 1,20–7,00; p = 0,017) and absence of clinical nodal metastases (OR 3,10, 95% CI 1,45–6,55; p = 0,003) remained significant (**Table 1**). Indeed, when building a (ROC) for the probabilities obtained from this model, an area under curve (AUC) of 0,69 (95%CI: 0,6 – 0,78; p < 0,0001) was obtained. With an optimal cut-off point of 0,42, sensitivity of 72,6% and specificity of 51,6% was obtained in predicting T feature downstage post-NET.

Other factors, such as ER, PR and Ki-67 were also tested, but did not reach statistical significance (data not shown).

### 3.2 Nodal response

**Figure 1:**
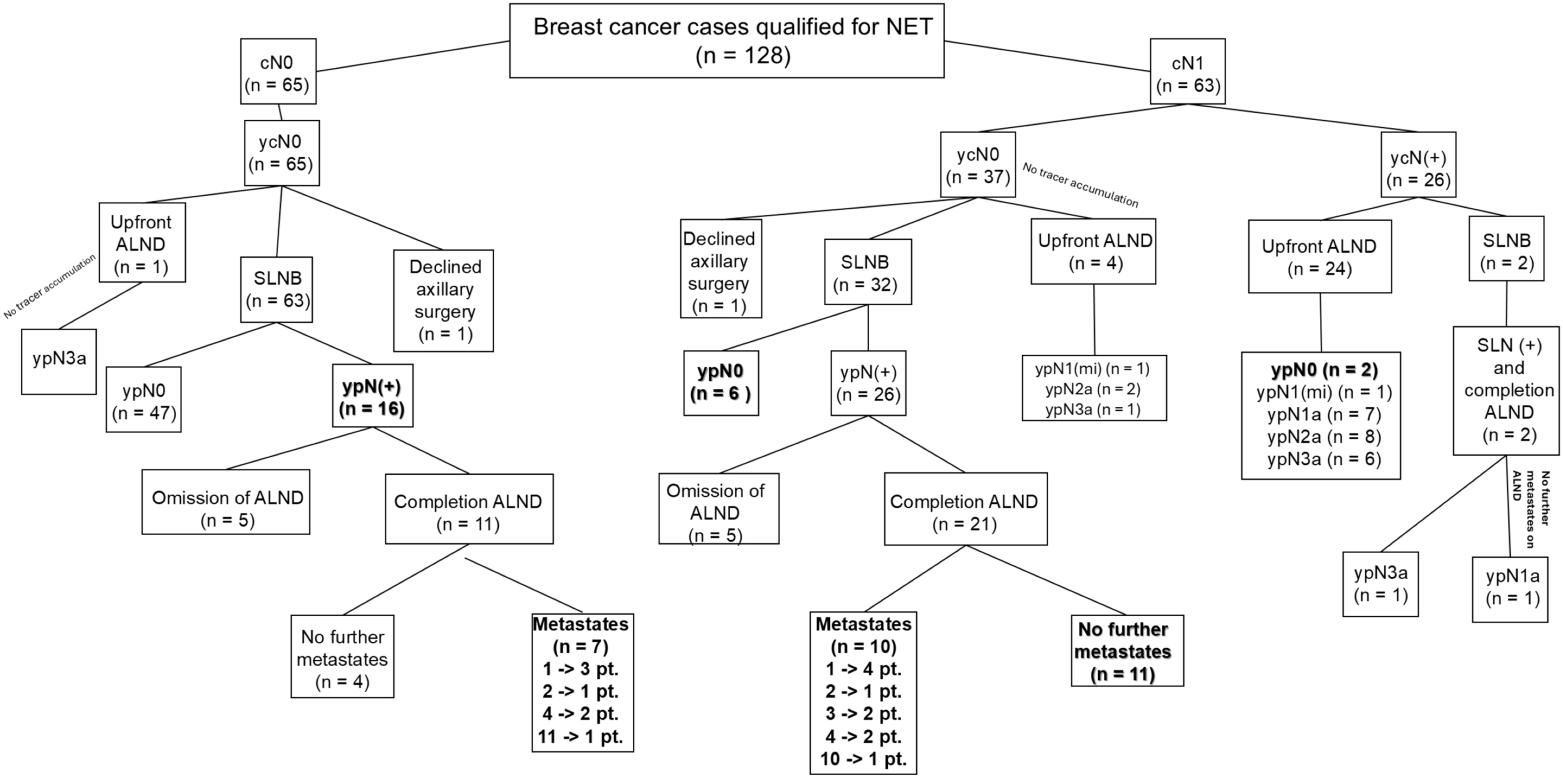
The flow chart of the surgical management of axilla and outcomes.

The analyzed group consisted of 65 cN0 cases (50,78%), 60 cN1 cases and 2 cases and 1 case of cN2 and cN3, respectively (**Table 5**). Last clinical and radiological examination prior to surgery revealed 102 cases of ycN0, 25 cases of ycN1 and 1 case of ycN2. Two patients declined nodal surgery. Twenty-four patients were then qualified to upfront ALND and the rest of patients were qualified to upfront SLNB. The flow chart of nodal surgery and response is shown in **Figure 1**, whereas **Table 6** summarizes lymph node yield and pathological involvement after SLNB and ALND.

**Table 5:** Distribution of N feature in study population.

Pretreatment clinical nodal stage				
cN0	cN1	cN2	cN3	
65	60	2	1	
Post-neoadjuvant clinical nodal stage				
ycN0	ycN1	ycN2	ycN3	
101	25	1	0	
Post-neoadjuvant pathological nodal stage				
ypN0	ypN(mi)	ypN1a	ypN2a	ypN3a
55	6	36	17	12

**Table 6:** Infiltration of lymph nodes on histopathological examination.

Type of lymph nodes	Mean (median) +/- SD
SLNB nodes infiltrated	0,83 (0) +/- 1,16
SLNB total nodes harvested	3,6 (3) +/- 1,6
ALND nodes infiltrated	4,33 (2) +/- 6,35
ALND total nodes harvested	15,6 (15) +/- 6,88
Total macrometastates	2,32 (1) +/- 4,17
Total micometastates	0,54 (0) +/- 1,1

#### 3.2.1 cN0 group

In clinical node-negative disease group, all 65 patients were deemed node-negative in the last clinical and radiological examination prior to surgery. Thus, all patients were initially qualified to SLNB. One patient was intraoperatively converted to ALND, as no tracer accumulation was present in lymph nodes. This patient proved to be ypN3a on histopathological examination.

Remaining patients had SLNB carried out- there were 16 cases of SLN (+) [25,4% of cases]. In 5 cases (7,8% of SLNB cases), after a detailed discussion with patients, during a multidisciplinary tumor board, a decision on ALND omission was reached. All those patients had BCS and they were scheduled for breast and axillary radiotherapy. In the remaining 11 cases (17,2% of SLNB cases), a completion ALND was performed. In 4 cases (36,3% of completion ALND cases) no further metastases where found on histopathological examination. There were three cases (27.3% of completion ALND cases) with a single macrometastasis. These findings corresponded to two patients with a total of two macrometastases and one patient with a total of three macrometastases. In the remaining completion ALND cases, the number of metastatic lymph nodes was 2 (n = 1), 4 (n = 2), and 11 (n = 1), respectively.

#### 3.2.2 cN1 group

In the last examination of cN1 cases, prior to the surgery, there were 37 cases of ycN0 (58,73%) and 26 cases of ycN(+) disease (41,27%) (**Figure 1**). In ycN(+) group two patients had SLNB due to lack of palpable nodes and ambiguous morphology of the nodes on ultrasound and patients’ preference. Yet, both cases required completion ALND due to SLN(+). One of above mentioned patients proved to be ypN3a, while the other one was ypN1a, as there were no metastases in completion ALND specimen.

The remaining 24 cases of ycN+ (92,3%) patients had an upfront ALND. In this group there were 2 cases of nodal pCR (8,3%), one case of micrometastates, while the other cases had more extensive nodal involvement.

36 cases of ycN0 were qualified for SLNB. Four of them were intraoperatively converted to ALND, as no tracer accumulation was present (**Figure 1**). Nodal involvement (of at least ypN1(mi)) was present in all of these cases. In the other cases, there were 6 cases of nodal pCR (18,75%) and 26 cases of SLN(+) (81,25%). Twenty one patients were scheduled for completion ALND, while 5 patients opted for ALND omission after detailed discussion during MDT meeting. In the ALND completion group, there were no further metastases in 10 cases (47,6%).

#### 3.2.3 Summary of nodal surgery results and future perspective

There were overall 8 cases of complete pathological nodal response (12,7% of cN(+) cases). Additionally, there were 4 cases of residual nodal disease presenting as one micrometastasis (6,35% of cN(+) cases).

The correlation analysis showed that a moderately strong positive correlation between ER stain and number of infiltrated nodes (rho = 0,21; p = 0,02). Patients with ER stain of 100% had on average 3,11 infiltrated nodes while in case of ER stain below 100% this average amounted to 1,35 (p = 0,017).

A further analysis was performed in order to identify factor associated with complete pathological nodal response. In the logistic regression analysis the only factor associated with the higher odds of complete pathological nodal response was PR expression between 31% and 60% (OR: 8 95%CI: 1,58 – 40,6; p = 0,012). The analysis of potential factors is shown in **Table 2**.

**Figure 2:**
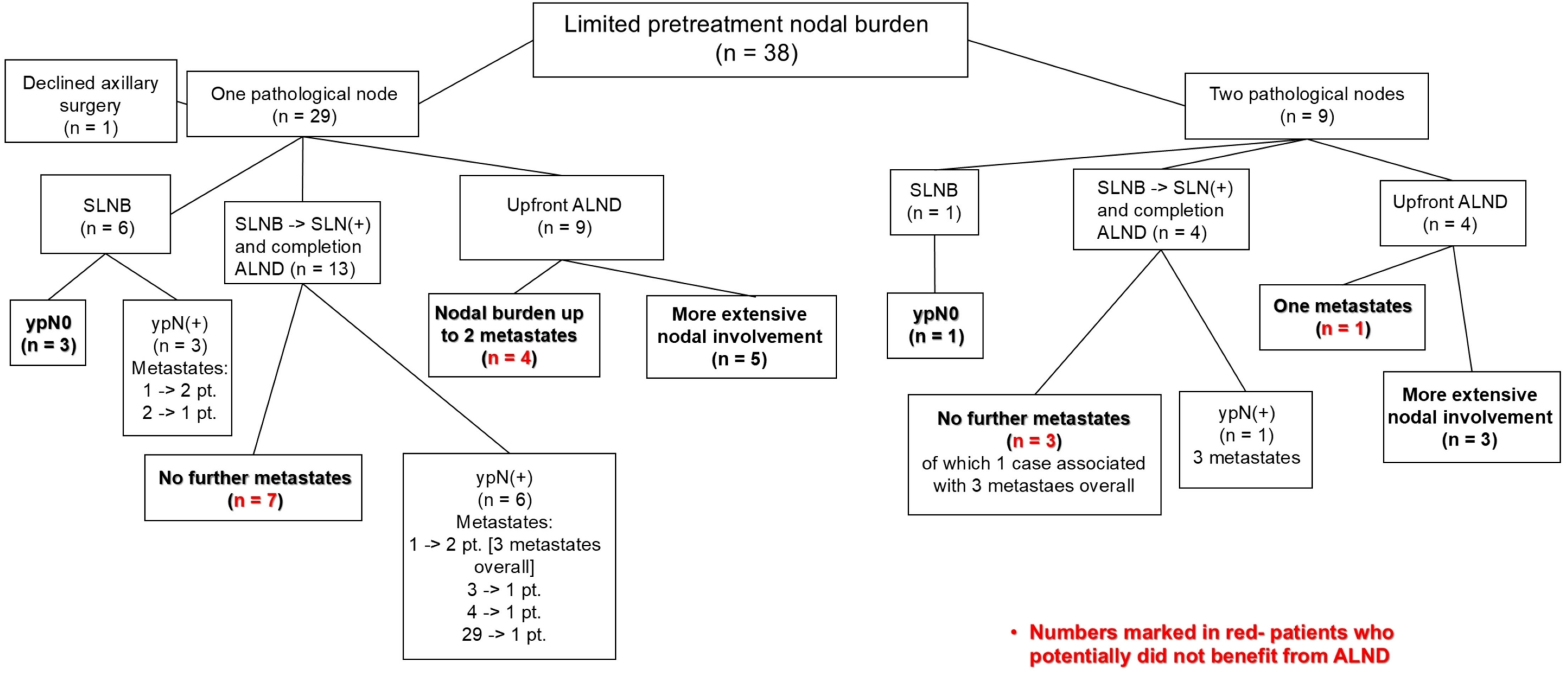
The flow chart of the surgical management of axilla and outcomes in patients with limited nodal burden.

Considering the recent recommendations on upfront surgery in luminal breast cancer with 1–2 clinically positive lymph nodes on preoperative imaging, confirmed by needle biopsy (36), patients in our study group with 1–2 clinically positive lymph node on imaging were also analyzed separately. Namely, our subgroup consisted of 29 cases with one clinically positive axillary lymph node, 9 cases with two clinically positive lymph nodes. The flow chart of axillary management in those patients is depicted in **Figure 2**.

In group of 29 patients with one clinically positive node (cN1 prior to NET), one patient declined axillary surgery, 9 patients underwent upfront ALND, 13 cases were qualified for completion ALND after SLN(+) was revealed in SLNB specimen and 6 underwent only SLNB. In SLNB group 3 patients had pCR, while 1 and 2 infiltrated nodes, where revealed in 2 and 1 cases respectively but ALND omission decision was reached during MDT. In upfront ALND group, in 4 cases there was nodal burden up to 2 macrometasates, while in remaining 5 cases more extensive disease was diagnosed. In the completion ALND group, 7 patients had no further metastases, 2 patients had only one infiltrated node (however both patients had 2 metastases in SLNB), while in the other 4 cases ALND specimen revealed 3, 4 and 29 infiltrated nodes.

In group of 9 patients with two clinically positive lymph nodes, one patient underwent SLNB, 4 cases were qualified for ALND, while completion ALND after SLNB was performed in 4 cases. Patient undergoing SLNB had pCR, while in the ALND group one patient had one metastasis and in the remaining cases more extensive nodal involvement was present. In the ALND completion group 3 patients had no further involvement (however one case was associated with 3 metastases in SLNB) and one patient had additional 3 metastases.

## 4 Discussion

Despite intense research into the optimal management of HR+/HER2- breast cancer, especially in case of limited nodal disease, the role of neoadjuvant therapy and upfront surgery is still not clearly established. While NET can be considered to facilitate BCS when no clear indications to chemotherapy are present (37, 38), the duration of the therapy and potential side effects are main disadvantages to regard. These recommendations mostly apply to postmenopausal women, as NAC is favored in premenopausal patients. Indeed, studies show better response rate and lower all-cause mortalities in premenopausal women treated with NAC (39, 40). It should be noted, however, that response rate to NET in general population of patients is also high (41) with some research suggesting similar response rate to NAC and NET (42, 43).

One of the main issues associated with the decision of implementation of NET or NAC is lack of proper prognostic tools that could facilitate the selection of patients that would benefit from this treatment in terms of tumor and nodal response. There are ongoing studies, however, that could facilitate tailoring the neoadjuvant regimen. There is evidence that gene assays can identify patients that would benefit from NET rather than from NAC basing on their genomic risk (44). Moreover, performing multigene panel- OncotypeDX^©^ Recurrence Score (RS) on core biopsy proved to be useful in predicting patients’ response to NET (45). Other commonly utilized gene expression signatures such as MammaPrint, BluePrintPAM50, EndoPredict, and the Breast Cancer Index have been studied in context of response to neoadjuvant therapies, however the data is still limited (46). As for conventional markers, number of studies suggest that ER expression level and baseline Ki-67 expression can predict response to NET (47–50). In our cohort, however, both markers did not reach statistical significance in predicting pCR or either local or nodal response. We found however that positive PR stain between 31% and 60% correlated with complete nodal response. Its molecular mechanism might be explained by interplay between ER and PR receptors under physiological circumstances. The imbalance in ER and PR receptors expression can likely reflect cellular instability and abnormalities in signaling pathways (51, 52). Nevertheless, this observation requires further studies, as there is still limited evidence to support it. Study by Collins et al. explored PR score in setting of neoadjuvant chemotherapy (53). They concluded that lower PR score was independently predictive of downstaging following NAC, but PR positivity also independently predicted increased risk of pathological nodal involvement.

Regarding the histopathological subtype of cancer, currently there is no clear evidence as to whether ductal or lobular carcinoma responds better to NET. It should be noted that in a retrospective study of circa 6000 cases of node-positive lobular breast cancer, NET was associated with similar survival but lower mastectomy rates than neoadjuvant chemotherapy (54).

Preoperative NET should not be used in patients in whom primary breast-conserving treatment (BCT) is possible with acceptable aesthetic results, in whom NET will not change the extent of the procedure, e.g. due to extensive microcalcifications, or in patients who are eligible for mastectomy after HT.

As for retrospectively acquired clinical data, the analysis of axillary surgery in our cohort seems particularly interesting. Taking into account the recent publication on tailored axillary surgery the gathered data supports the notion that patients with 1–2 pathological lymph nodes on preoperative imaging should rather undergo upfront surgery with targeted axillary surgery. The odds of complete nodal response with NET is indeed low, so proper staging of axilla in upfront surgery seems more appropriate, as significant portion of patients who underwent ALND or completion ALND did not benefit from procedure in light of the recent studies (55, 56). On the other hand, it should be noted that there is some risk of understaging of axillary disease when ALND is not performed. Indeed, in our cohort, a relatively big fraction of patients had more extensive lymph node infiltration in ALND specimen. A similar conclusion was recently draw in a study within OPBC-03/TAXIS trial (57). Nonetheless, authors’ analysis proved that ALND did not inform systemic therapy recommendations. Study by Peery et al. (58) highlighted a trend towards ALND omission in ypN(+) patients receiving either NAC or NET. In NET subgroup, 28,5% of ypN+ patients did not undergo ALND. The oncological safety of this approach was not yet reported in either of study, therefore the results of longer follow-up in prospective trials are crucial to support such a management.

The results of our study should be interpreted in the context of a broader paradigm shift toward individualized and de-escalation-oriented axillary management. In a recent prospective, multicenter clinical trial, Mamtani et al. (59) demonstrated that upfront SLNB is feasible in patients with cN1 HR+/HER2− breast cancer selected based on limited nodal burden on axillary ultrasound, with nearly 70% of patients having only 1–2 positive nodes and successfully avoiding ALND. Importantly, the study by Mamtani et al. challenges the traditional paradigm that clinically node-positive disease uniformly requires ALND, demonstrating that more than half of such patients may have limited nodal involvement amenable to less extensive surgical management.

Moreover, recent update on NCCN guidelines (36) recommends omission of upfront ALND in favor of SLNB in limited axillary lymph node burden on imaging, confirmed by needle biopsy. It should be noted that NCCN recommends ALND omission only in patients with no palpable lymph nodes while in the above-mentioned study patients with palpable adenopathy were enrolled. In fact, another recent study by Cardarelli et al. (60) examined 57,823 patients from National Cancer Database. They found no difference in OS for patients staged as cN0 versus cN1 who were found to be pN1. Their results indicate thus that sole assessment of lymphadenopathy can lead to overstaging and consequently to unnecessary ALND. Other retrospective study of patients from National Cancer Database (61) also indicated that omission of ALND in limited nodal burden does not negatively impacts OS, as cN(+) patients undergoing SLNB + regional nodal irradiation had non-inferior results to those undergoing ALND with or without regional nodal irradiation. It should be noted, however, that the mean follow-up duration in both aforementioned studies may be too short to fully assess the impact of ALND omission on OS or DFS.

Taking into account this evidence, 14 out of 30 patients from our study (46.67%), who eventually underwent ALND potentially did not benefit from this treatment, as only up to two metastases were present in the axilla.

It is also worth considering whether patients with luminal HER2-negative breast cancer who have undergone NET of relatively short duration (6–8 months) should be managed in the same way as patients treated with neoadjuvant systemic therapy (e.g. TNBC or HER2-positive disease), particularly with regard to qualification for ALND in the presence of ypN+ findings in SLNB. These patients will, after all, receive long-term post-neoadjuvant endocrine therapy, most often in combination with CDK4/6 inhibitors.

In summary, NET is a vital, well-tolerated neoadjuvant approach in selected postmenopausal HR+/HER2− breast cancer patients. By optimizing patient selection, incorporating predictive biomarkers like Ki−67 and/or Ki-67 dynamic response to NET (62–64), and tailoring treatment duration, NET offers a means to improve clinical outcomes and quality of life. As personalized oncology and de-escalation strategies evolve, NET deserves broader clinical integration.

## Data Availability

The raw data supporting the conclusions of this article will be made available by the authors, without undue reservation.
